# Global Deletion of TSPO Does Not Affect the Viability and Gene Expression Profile

**DOI:** 10.1371/journal.pone.0167307

**Published:** 2016-12-01

**Authors:** Huaishan Wang, Kangle Zhai, Yingchao Xue, Jia Yang, Qi Yang, Yi Fu, Yu Hu, Fang Liu, Weiqing Wang, Lianxian Cui, Hui Chen, Jianmin Zhang, Wei He

**Affiliations:** 1 Department of Immunology, Research Center on Pediatric Development and Diseases, Institute of Basic Medical Sciences, Chinese Academy of Medical Sciences and School of Basic Medicine, Peking Union Medical College, State Key Laboratory of Medical Molecular Biology, Beijing, China; 2 Beijing Thorgene Medical Laboratory, Yizhuang Biomedical Park, Beijing, China; 3 Department of Otolaryngology, Peking Union Medical College Hospital, Chinese Academy of Medical Science and Peking Union Medical College, Beijing, China; Cornell University, UNITED STATES

## Abstract

Translocator Protein (18kDa, TSPO) is a mitochondrial outer membrane transmembrane protein. Its expression is elevated during inflammation and injury. However, the function of TSPO *in vivo* is still controversial. Here, we constructed a TSPO global knockout (KO) mouse with a Cre-LoxP system that abolished TSPO protein expression in all tissues and showed normal phenotypes in the physiological condition. The birth rates of TSPO heterozygote (Het) x Het or KO x KO breeding were consistent with Mendel’s Law, suggesting a normal viability of TSPO KO mice at birth. RNA-seq analysis showed no significant difference in the gene expression profile of lung tissues from TSPO KO mice compared with wild type mice, including the genes associated with bronchial alveoli immune homeostasis. The alveolar macrophage population was not affected by TSPO deletion in the physiological condition. Our findings contradict the results of *Papadopoulos*, but confirmed *Selvaraj*’s findings. This study confirms TSPO deficiency does not affect viability and bronchial alveolar immune homeostasis.

## Introduction

Translocator Protein (18kDa) (TSPO), previously known as Peripheral Benzodiazepine Receptor (PBR), is an outer mitochondrial membrane protein with five transmembrane domains[1–3]. TSPO is widely expressed in different tissues including the adrenal cortex, white adipose tissue, brown adipose tissue, lung, liver, spleen and thymus[4,5]. However, its precise function remains unclear. Several inflammatory diseases are associated with elevated TSPO expression[6–8]. Increased uptake of radioisotope labeled high affinity ligands of TSPO have been used clinically as biomarkers to monitor inflammatory status in neurodegenerative diseases, brain injury and cancer[6,8–10]. Before TSPO knockout (KO) mice were constructed, knowledge regarding TSPO function was acquired from studies based on the hypothesis that TSPO is activated by ligands[8]. However, functions of TSPO *in vivo* are not clear.

TSPO deficiency was thought to be lethal during embryonic development given its presumed crucial roles in steroid biosynthesis, mitochondria functions and secondary signal transduction[8,11] However, *Selvaraj* and colleagues reported that TSPO KO mice survived with normal phenotypes[12]. Two independent research groups reported that TSPO KO mice survived to adulthood without overt phenotypes, developmental defects or cholesterol metabolism disorders[4,13]. In addition, *Papadopoulos* and colleagues reported that TSPO deficiency led to reduced viability, but *Selvaraj* suggested conflicting results[14]. These studies address the questions regarding what roles TSPO play during development.

In this study, we generated TSPO floxed mice using a Cre-LoxP system[15,16], with whole body deletion of TSPO. Consistent with previous studies[4,12,17], TSPO KO mice survived until adulthood with a birth rate consistent with Mendel’s Law. We know lung tissue is quiescent with unique immune homeostasis and high expression of TSPO, especially in bronchial alveolar epithelial cells and alveolar macrophages[7]. Our Digital Gene Expression Profiling (DGE) analysis showed no significant difference in the transcriptome profile of lung tissue between TSPO KO mice and wild type (WT) mice. This suggests that TSPO KO mice have normal gene expression profiles and normal bronchial alveolar immune homeostasis.

## Reagents and Methods

### Mice

All animal procedures were approved by the Animal Care and Use Committee of Institute of Basic Medical Sciences, Chinese Academy of Medical Sciences (IBMS, CAMS). TSPO Floxed mice, Flp transgenic mice and Protamine-Cre transgenic mice were C57BL/6J background and mice were maintained in SPF conditions at Experimental Animals Center of IBMS-CAMS. Mice were euthanized by Carbon Dioxide, for tissue isolation, mice were anesthetized by sodium pentobarbital.

### Genotyping

TSPO floxed and KO pups were labeled by cutting toes 7 to 10 days after birth, one toe or tail tip was selected for genotyping. Genomic DNA was isolated according to the Dirty DNA Isolation Protocol of the Jackson Laboratory[18]. Briefly, samples were digested 90min at 98°C, placed at 15°C with 75μl NaOH-EDTA solution (25mM NaOH, 0.2mM EDTA), then mixed with 75μl 40mM Tris-HCl (pH5.5) Genomic DNA was used for genotyping. Standard PCR (Taq Master Mix, Quick Taq HS DyeMix, TOYOBO) was used, annealing temperature was 60°C with 35 cycles. The oligonucleotides for mouse genotyping were Flp forward (F) = 5’-tac aag tgg atc gat cct acc cct tgc g-3’; Flp reverse (R) = 5’-tcc cag gtc caa ctg cag ccc aag ctt cc-3’; Protamine-Cre F = 5’-CAT GTT CAG GGA TCG CCA GGC GTT T-3’; Protamine-Cre R = 5’-GTG CTA ACC AGC GTT TTC GTT CTG CCA A-3’; KO Forward (P1) = 5’-GAT GGA GAA ACT GAG TCC CAG TCA GGG T-3’; KO Reverse (P2) = 5’-GCT CTG CCC TAA TCA CAA AGT TTC ACA C-3’; KO Reverse (P3) = 5’-TTA AGG AGA GGT TTT GTC CTT GTG TC-3’.

### Antibodies

Anti-mouse TSPO Antibody (#9530) for Western blot was purchased from Cell Signaling Technology^®^ (CST, Danvers, Massachusetts); Anti-PBR (TSPO) RabMAb^®^ [EPR5384] for WB and IHC was purchased from Abcam (Cambridge, UK); Mouse Anti-GAPDH antibody was purchased from BOSTER (BM1623, Wuhan, P.R.China); Alexa Fluor^®^ 488 anti-mouse F4/80 Antibody (BM8) and Alexa Fluor^®^ 647 anti-mouse CD206 (MMR) Antibody (C068C2) were purchased from BioLegend^®^ (San Diego, California, U.S.); HRP anti-mouse/rabbit IgG secondary antibodies for WB and DAB Kit for IHC were purchased from ZSGB-BIO (Beijing, P.R.China); Mouse on Mouse (M.O.M.^™^) ImmPRESS^™^ HRP (Peroxidase) Polymer Kit and ImmPRESS^™^ HRP Anti-Rabbit IgG (Peroxidase) Polymer Detection Kit for IHC were purchased from Vector Laboratories, Inc.(Burlingame, Ca, U.S.).

### Western blotting

TSPO expression of different tissues was examined by Western blot as previously described[19]. Tissues were homogenized in EDTA-free RIPA buffer containing protease inhibitor (Thermo Scientific) on ice, lysates were separated through 12% SDS-PAGE and blotted onto NC membrane. After incubating TSPO antibody (CST) for 36h-48h at 4°C and HRP-secondary antibody for 1h at room temperature, signal was exposed with Supersignal West Pico Chemiluminescent Substrate (Thermo Scientific) and CliNX chemiscope 3400 (CliNX Science Instruments Co., Ltd).

### H&E staining and immunohistochemistry (IHC)

TSPO WT and KO (6 weeks, male) were anesthetized with 2% sodium pentobarbital (100mg/kg), and then fixed by cardiac perfusion with 4% PFA and processed for H&E staining and IHC as described previously[4]. Briefly, tissue sections were blocked with 5% goat serum and incubated with Anti-PBR (TSPO) RabMAb^®^ (Abcam, 1:1500) at 4°C overnight and ImmPRESS^™^ HRP Anti-Rabbit IgG (Peroxidase) Polymer Detection Kit at room temperature for 45 minutes. HRP was detected with DAB Kit (ZSGB-BIO). Nikon microscope was used for whole viewed section scan.

### RNA-seq and differential analysis

Total RNAs from lung tissues of 9 TSPO WT and 9 TSPO KO mice were isolated, 3 samples of every group were combined after RNA extraction for quality control and RNA-seq loading samples. RNA-seq was performed as DGE on an Illumia HiSeq platform and 50 bp paired-end reads were generated (RiboBio Co. Ltd.). The NCBI Gene Expression Omnibus (GEO) of the deep sequencing data were submitted under accession umber GSE84942. High-quality reads were aligned to the mouse reference genome (mm10)[20]. Significant differences were analyzed by negative binomial distribution test after read-counts normalization[21], fold change and adjusted *P* value (Q value) by the Benjamini-Hocherg false discovery rate (FDR) procedure (Q value) were considered as factors for differential analysis[22]. Genontology (GO) and Kyoto Encyclopedia of Genes and Genomes (KEGG) pathway analysis were performed as previously described[22–24]. DAVID (NIAID, NIH) Database was employed for gene annotation analysis[25,26]. TSPO WT and KO locus (mm10) of RNAseq were analyzed by IGV[27,28] software. Heat maps of interaction protein genes and bronchoalveolar immune microenvironment associated genes were analyzed by Multiple Array Viewer (MeV) v4.9 software[29].

### Bronchoalveolar lavage fluid (BALF)

Mice were anesthetized, trachea was exposed, BALF and bronchovesicular cells were collected by lung lavage twice with 1ml normal saline containing 0.05% EDTA. BALF was centrifuged at 4,500 rpm, the first lavage supernatant was used to detect cytokines and mixed cells for flow cytometry analysis.

### Flow cytometry

Flow cytometry of alveolar cells was preformed as previously described[30]. BALF cells were resuspended in wash buffer (1% BSA-PBS), fluorescently labeled antibodies were incubated at 4°C in dark for 20 minutes, and washed twice with ice wash buffer. Cells were detected by BD C6 Flow Cytometry and the data were analyzed by C6 software.

### Statistical analysis

Results are presented as mean ± S.E.M. unless otherwise specified. Statistical significance was determined by Student’s *t*-tests; Birth rate difference compared with Mendel’s law was detected using Chi-squared test. Differences of statistical analysis at *P* < 0.05 were considered significant.

## Results

### Generation of TSPO KO mice

**Fig 1:**
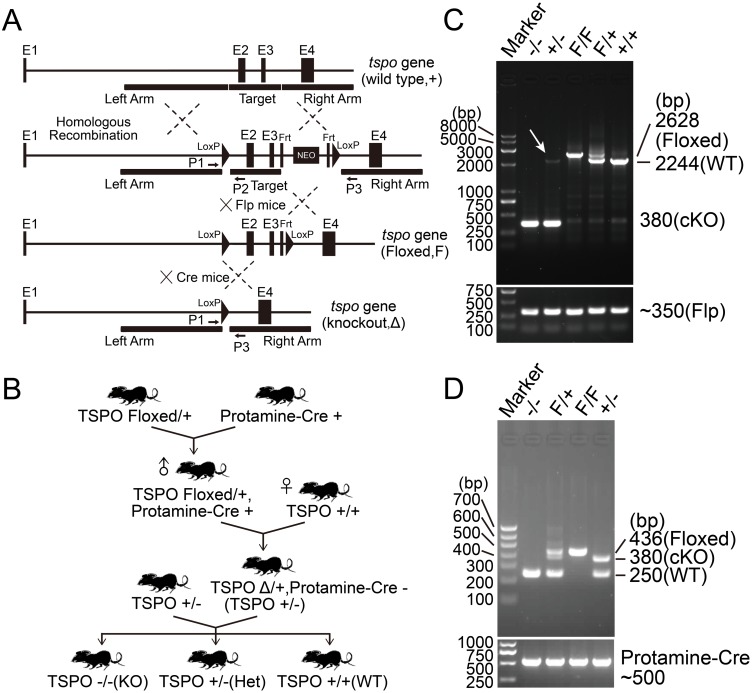
Generation of TSPO KO mice. (A) Schematic of generation of TSPO Floxed mice and conditional KO mice by Cre-LoxP System. Conditional TSPO KO mice were generated by targeting exon 2 and exon 3 of the mouse genomic locus, a NEO selection cassette Floxed by Frt sites. (B) Schematic of crossing program for TSPO global knockout mice, Protamine-Cre is a transgenic mouse where Cre recombinase expression is driven by sperm specific protamine promotor. (C) Genotyping of TSPO Floxed mice and KO mice by a TSPO genotyping primer pair 1 & 3, and Flp primers. (D) Genotyping of TSPO Floxed mice and KO mice by three TSPO genotyping primers 1, 2 and 3, and Protamine-Cre primers.

Given previous evidence that deficiency could be lethal during embryonic development[11], TSPO floxed mice for conditional deletion were generated by targeting the transmembrane domains contained within exon 2 and exon 3 of the mouse genomic locus. TSPO targeting vector was constructed by introducing two LoxP sites between exon 2 and exon 3 (Target region) and a NEO selection cassette floxed by Frt sites (Fig 1A). TSPO was genomically deleted by breeding with the male germ-line protamine-Cre deleted mice. After two rounds of breeding, heterozygote mice were generated and then intercrossed to generate homozygote TSPO KO mice (Fig 1B). TSPO KO mice were confirmed by PCR (Fig 1C and 1D). The homozygote TSPO KO mice were viable to adulthood, consistent with previous findings[4,12].

### TSPO KO mice have normal viability

**Fig 2:**
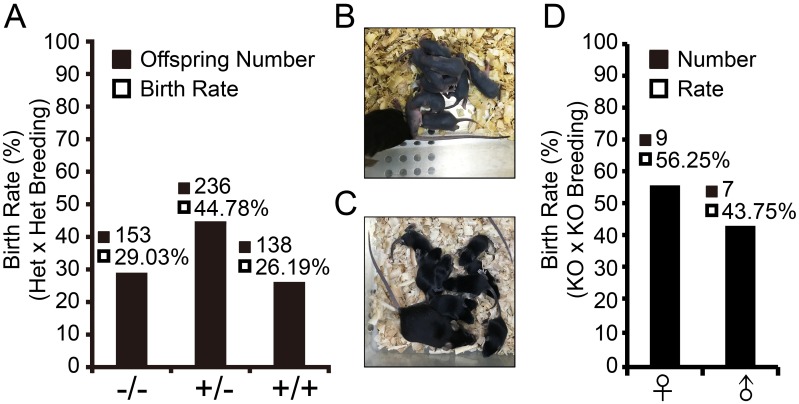
TSPO deletion does not affect viability by Het x Het and KO x KO breeding. (A) Birth rate of offspring of TSPO Het x Het breeding. (B) One litter Pups born 4 days from TSPO KO x KO breeding. (C) One litter pups born 9 days from TSPO KO x KO breeding. (D) Birth rate of offspring in TSPO KO x KO breeding. Chi-Squared Test was used to determine significant difference compared with Mendel’s Law.

To determine the viability of TSPO KO mice, TSPO Het x Het mouse breeding was set up to examine the birth rate of each genotype (WT, Het and KO). 527 offspring were born during more than 2 years of Het x Het breeding and consisted of 138 WT (26.19%), 236 Het (44.78%) and 153 KO (29.03%) mice (Fig 2A). These numbers were consistent with Mendel’s Law: WT 25%, Het 50%, KO 25% and 50% female, 50% male. No significant difference was observed in the birth rates by Chi-Squared Tests (*P*<0.05). Thus, TSPO knockout did not affect the viability of mice. To further verify the viability rate of TSPO KO mice, TSPO KO x KO mouse breeding was also set up. Two litters produced 16 pups with normal physical condition (Fig 2B and 2C) and gender birth rate (Fig 2D).

### Global deletion of TSPO did not affect the morphology of main organs and tissues

**Fig 3:**
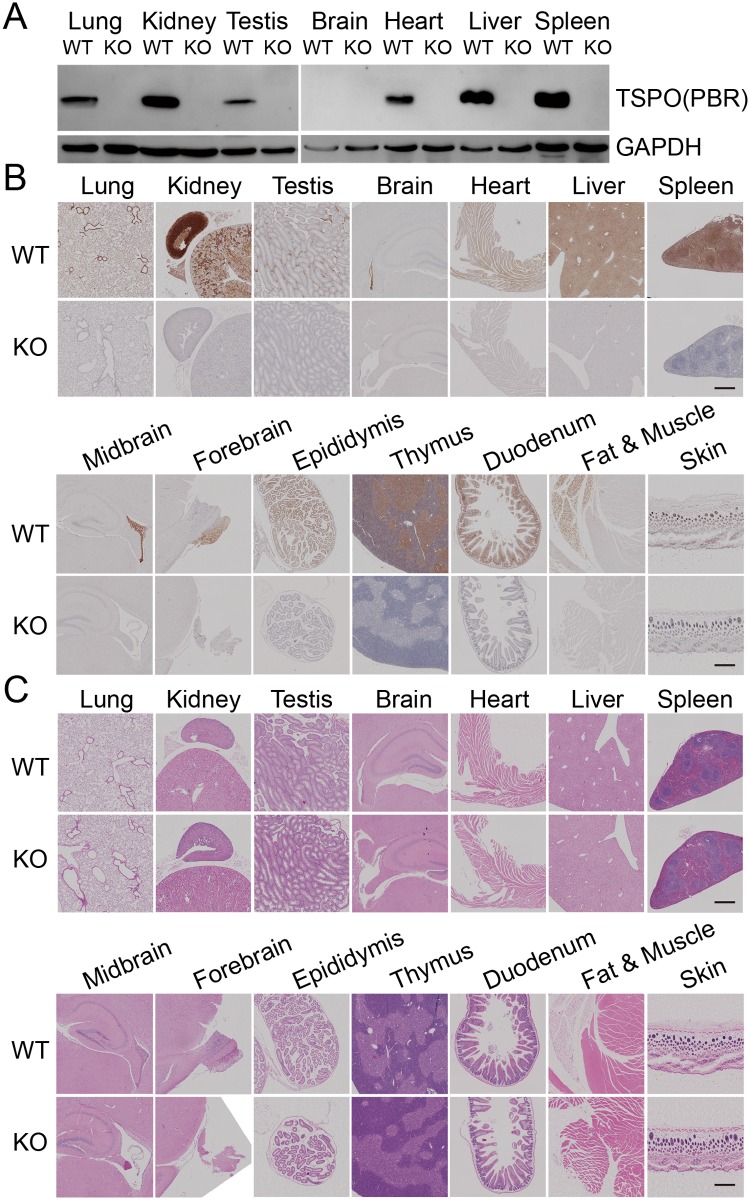
TSPO expression was abolished in global KO mice without pathological changes. TSPO expression in different tissues from WT and KO mice were detected by western blotting (A) and IHC (B); (C) H&E staining of different tissues from WT and TSPO KO mice. Scale Bars, 100μm.

Previous studies demonstrated that TSPO KO mice had normal phenotypes[4,12,13,17]. To further characterize the phenotypes of TSPO KO mice, we first examined the expression of TSPO protein in major organs and tissues from TSPO WT and KO mice by Western blotting and IHC. The results show high levels of TSPO expression in the bronchial alveolar epithelium, alveolar macrophages, heart muscle, liver, spleen, thymus, kidney, testicular/epididymal mesenchymal, fat, skin, duodenum, ependyma and olfactory bulb. In comparison, we found low levels of TSPO expression in lung parenchyma, brain cortex, hippocampus, midbrain and leg muscle tissue (Fig 3A and 3B). The expression of TSPO protein was completely deleted in all tissues in KO mice (Fig 3A and 3B). To determine whether global deletion of TSPO results in the morphological changes in the main organs or tissues, H&E staining of tissue sections from WT and TSPO KO mice was performed. No distinguishable morphological changes were observed in the main tissues from KO mice compared with WT mice (Fig 3C). These results suggest that whole body deletion of TSPO does not affect the development of major organs and tissues in TSPO KO mice.

### TSPO deletion did not affect gene expression profile in lungs of TSPO KO mice

**Fig 4:**
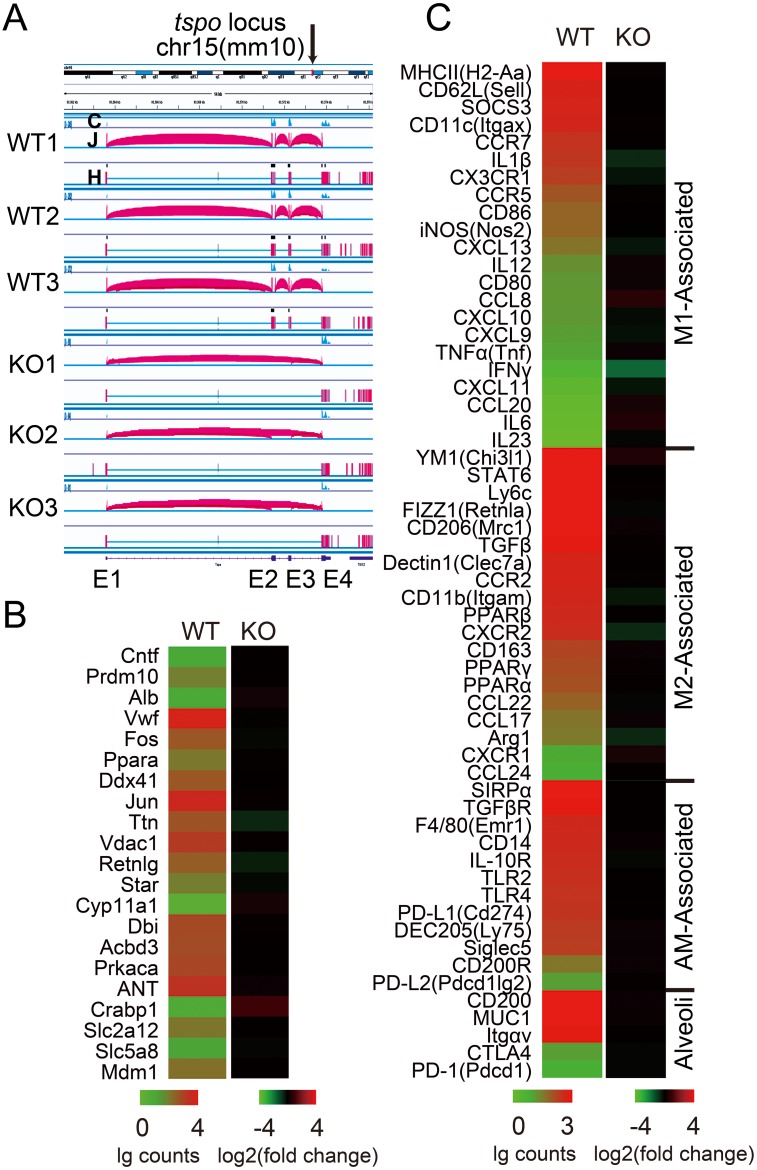
TSPO KO did not affect gene expression profiles. (A) TSPO gene locus of WT and KO mice lung tissue samples used for RNA-seq, results visualized by Integrative Genomics Viewer (IGV) software (NIH), C: coverage of reads, J: junctions, H: hits; (B) Expression level of potential TSPO interaction proteins reported by literatures and SRING database (Version 10.0); (C) Expression profile of bronchoalveolar immune microenvironment associated genes; (B&C) Significant difference determined by negative binomial distribution test.

To ascertain which genes were impacted after TSPO deletion, transcriptome profiles of WT and KO lung tissues were analyzed using RNA-seq. The mouse TSPO gene is located at chr15:83,561,573–83,576,203 (mm10). Fig 4A shows the locus of samples for RNA-seq by IGV software. TSPO KO mouse model was constructed by deleting exon 2 and 3 (Fig 1A). RNA-seq data confirmed the deletion of this region (Fig 4A). TSPO interacting proteins reported in the literatures[31,32] and STRING (functional protein association networks) database (Version 10.0)[33] were analyzed and are presented as a heat map (Fig 4B). TSPO was the only differentially expressed gene between TSPO WT and KO mice (data not shown). We also focused on pulmonary alveolar epithelium and macrophage related genes, which are crucial regulators of the steady state of the alveolar immune microenvironment (Fig 4C). TSPO KO mice showed no difference compared with WT mice, indicating TSPO deficiency does not affect the expression of TSPO interacting proteins and bronchial alveolar immune microenvironment homeostasis (Fig 4B and 4C).

### TSPO KO mice showed normal alveolar macrophage population

**Fig 5:**
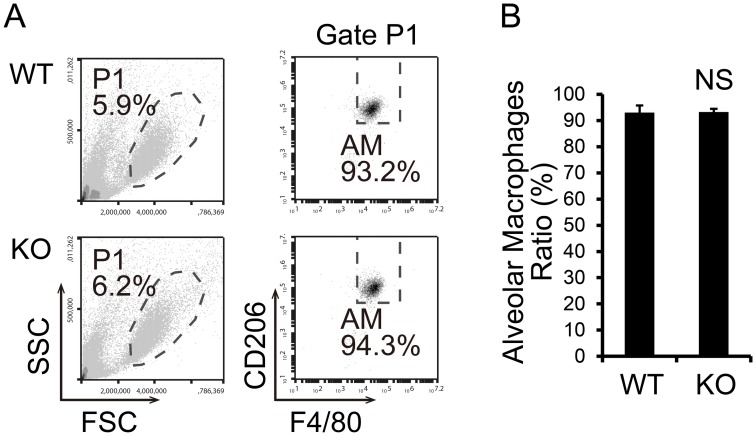
TSPO KO mice show normal alveolar macrophage population. (A) FACS analysis of BALF and alveolar macrophages was identified as F4/80+, CD206+. (B) The percentages of alveolar macrophages in BALF from WT and TSPO KO mice are shown as the mean ± S.E.M. from four animals in each group (n = 4). NS, not significant by Student’s *t*-test.

Alveolar macrophages are a specific macrophage subtype that reside in the alveolar duct in close contact with the respiratory epithelium. Mouse alveolar macrophages are classified as M2 macrophages and typically express F4/80, CD206, IL-10 receptor and TGFβ receptor[34]. We analyzed the alveolar macrophage population (F4/80+, CD206+) in bronchoalveolar lavage fluid (BALF) from WT and TSPO KO mice. The data show normal cell subtype populations in BALF and mainly alveolar macrophages in TSPO KO mice (Fig 5).

## Discussion

TSPO contains five transmembrane domains and is located at the outer mitochondrial membrane. Hundreds of previous studies demonstrated that TSPO possesses high affinity to cholesterol and plays a crucial role in the translocation of cholesterol from the cytosol to the mitochondrion[8,35–38], and is involved in the rate-limiting step in steroidogenesis[8]. Interestingly, recent *in vivo* studies based on knockout mouse models and *in vitro* studies based on deletion in steroidogenic cells have refuted the links between TSPO and cholesterol transfer[12,39]. The link between TSPO and mitochondrial energy homeostasis is inconsistent and perhaps dependent on cell type. Studies have reported that TSPO deletion results in a decreased oxygen consumption rate (OCR) in microglia[4] and fibroblasts[40], but no change in OCR was observed in hepatocytes[13] and Leydig cells[41]. An increase in mitochondrial fatty acid oxidation was observed in steroidogenic cells and tissues[41]. But TSPO is not required for steroid hormone biosynthesis as previously suggested[4,5,12,17,40]. The loss of TSPO in experimental autoimmune encephalomyelitis (EAE) mice, an animal model of multiple sclerosis (MS), caused mild astrogliosis and less EAE clinical scoring[42]. These studies demonstrated that TSPO KO mice exhibit defective phenotypes under pathological conditions. Inflammation and injury-derived TSPO upregulation was studied in multiple scenarios, including as an anti-inflammatory drug target, and as a positron emission tomography (PET) imaging radioligand target. The ligands of TSPO have been evaluated as protective agents to evaluate the inflammatory status in the brains of patients with Alzheimer’s Disease (PK11195 and Ro5-4864)[43], anxiety (XBD-173)[44], MS (PK11195)[45] and acute lung injury (ALI)[7].

In this study, we created TSPO knockout mice with the Cre-LoxP system and characterized the phenotypes under normal conditions to explore systemic physiological functions and networks of TSPO *in vivo*. In our study, the viability of TSPO KO mice was not affected in both Het x Het breeding and KO x KO breeding. Our results are consistent with *Selvaraj*’s findings[14], but contradict *Papadopoulos*[46]. RNA-seq analysis showed normal gene expression profile in lung tissue from TSPO KO mice. RNA-seq analysis showed that nearly all TSPO interacting proteins listed in the databases and published papers had normal mRNA levels in TSPO KO mice. These results indicate that TSPO does not play an important role in physiological conditions.

Several studies using knockout models both *in vitro* and *in vivo* have indicated that TSPO is not necessary for cellular and organismal survival. Recent evidences also suggest a strong disparity between pharmacological and genetic dissecting TSPO function. Several recent reviews have dissected these phenomena and explored possible functions for TSPO and its importance in cellular functions[47–51]. However, TSPO does play critical roles in biology, including endoplasmic reticulum associated protein degradation (ERAD), reactive oxygen species (ROS) production, cholesterol efflux, protoporphyrin IX (PPIX) synthesis, and even cytokine production, apoptosis and autophagy[52]. It is important to highlight two issues regarding TSPO. One is explaining why TSPO has a high affinity to cholesterol but does not participate in its transfer. The second is understanding why TSPO is overexpressed *in vivo*. A re-examination of the characteristics of TSPO with a transgenic mouse model is imperative, especially for drug target mechanism studies[8,44]. In future studies, using the “apparently healthy” knockout mouse model, we aim to uncover new features of TSPO that play roles in inflammation and neurodegenerative diseases.

## Additional Information

Deep sequencing data for the RNA-Seq experiments have been deposited in the NCBI Gene Expression Omnibus accession number GSE84942.
